# Supplementary material for: Ni-Catalyzed Enantioselective Intramolecular Mizoroki–Heck Reaction for the Synthesis of Phenanthridinone Derivatives
Source: J Org Chem. 2023 Jun 15;88(13):8203–26. doi: 10.1021/acs.joc.3c00202 (PMC10337041; doi:10.1021/acs.joc.3c00202)
Supplement: Supplementary file 2 — jo3c00202_si_002.zip [file jo3c00202_si_002.zip › Amides (N-MOM)/1e iPr-NMOM-ArBr/13C iPr-NMOM-ArBr 1e/1/pdata/1/email_DR-107-123 pure run 2 C13_1_1.pdf]

DR-107-123 pure run 2 C13
C13APT DMSO {D:\nmrusers\malachowski} BM 3

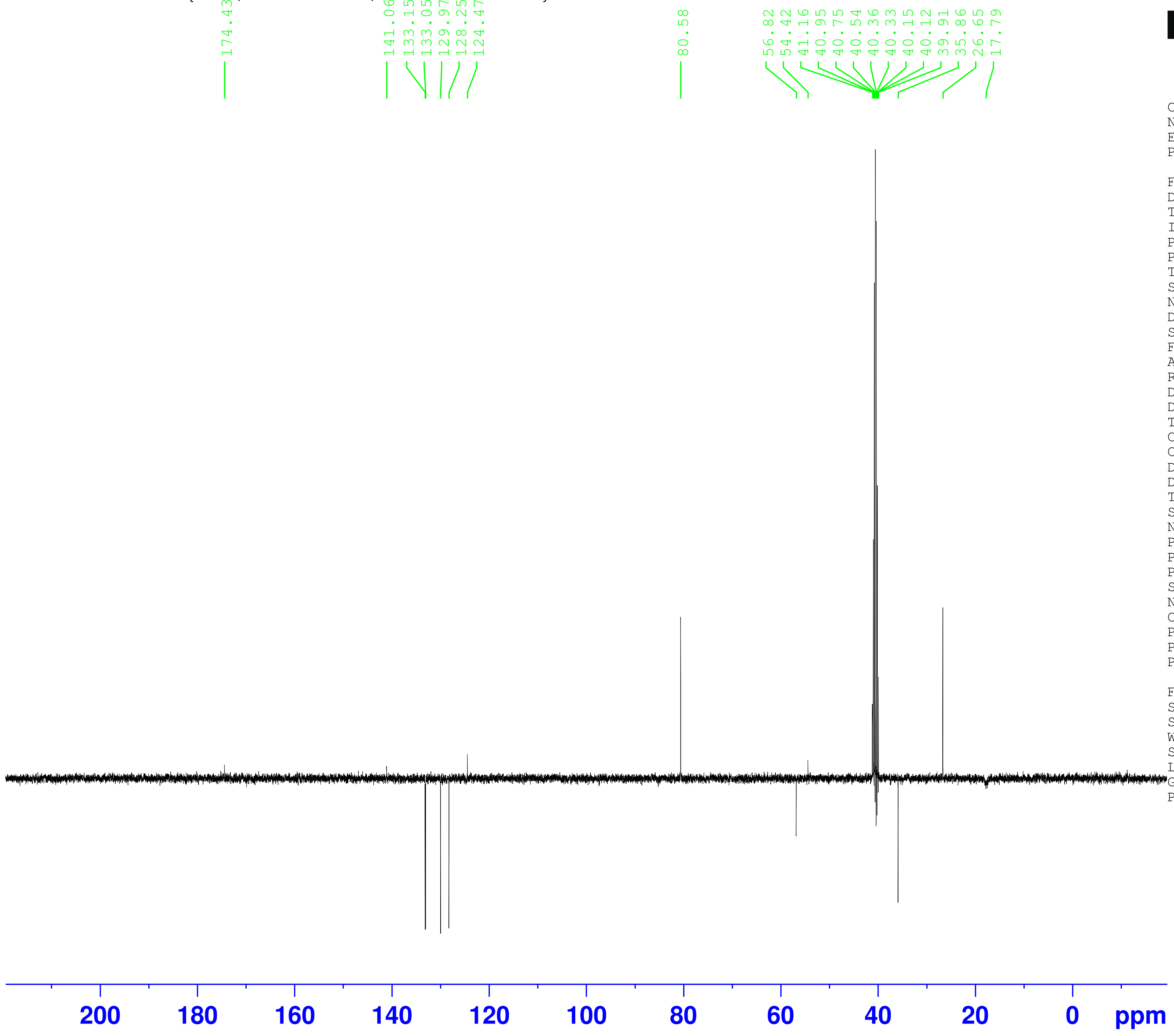

Current Data Parameters
NAME DR-107-123 pure run 2 C13
EXPNO 1
PROCNO 1

F2 - Acquisition Parameters

Date_	20210324	
Time	23.11	h
INSTRUM	spect	
PROBHD	Z104450_0352 (	
PULPROG	jmod	
TD	65536	
SOLVENT	DMSO	
NS	4000	
DS	4	
SWH	24038.461	Hz
FIDRES	0.733596	Hz
AQ	1.3631488	sec
RG	203	
DW	20.800	usec
DE	6.50	usec
TE	373.1	K
CNST2	145.0000000	
CNST11	1.0000000	
D1	2.00000000	sec
D20	0.00689655	sec
TD0	1	
SFO1	100.6278593	MHz
NUC1	13C	
P1	8.00	usec
P2	16.00	usec
PLW1	56.13299942	W
SFO2	400.1516006	MHz
NUC2	1H	
CPDPRG[2	waltz65	
PCPD2	90.00	usec
PLW2	12.14200020	W
PLW12	0.24368000	W

F2 - Processing parameters

SI	32768	
SF	100.6177975	MHz
WDW	EM	
SSB	0	
LB	1.00	Hz
GB	0	
PC	1.40	
